# The association of maternal smoking around birth with chronic respiratory diseases in adult offspring: A Mendelian randomization study

**DOI:** 10.18332/tid/189394

**Published:** 2024-06-27

**Authors:** Xiao-Jun Wang, Yun-Xia Huo, Wei-Dong Hu, Chaoyan Yue

**Affiliations:** 1 Department of Respiratory Medicine, Gansu Provincial Hospital, Lanzhou, People's Republic of China; 2 Department of Neurological Surgery, The Second People’s Hospital of Lanzhou City, Lanzhou, People's Republic of China; 3 Obstetrics and Gynecology Hospital of Fudan University, Shanghai, People's Republic of China

**Keywords:** maternal smoking around birth, adult offspring, chronic respiratory diseases (CRDs), mendelian randomization (MR) study, causal effect

## Abstract

**INTRODUCTION:**

Maternal smoking during pregnancy disturbs fetal lung development, and induces in their offspring childhood respiratory diseases. Whether it has a continued impact on offspring adult lung health and exerts a casual effect of chronic respiratory diseases (CRDs), remains uncertain. We seek to determine the causal relationships between maternal smoking around birth and offspring adult CRDs, using summary data from previously described cohorts.

**METHODS:**

Mendelian randomization (MR) study was used to analyze the genome-wide associations of maternal smoking around birth and offspring adult CRDs, including respiratory insufficiency, chronic obstructive pulmonary disease (COPD), related respiratory insufficiency, emphysema, COPD, COPD hospital admissions, early onset of COPD, later onset of COPD, asthma, idiopathic pulmonary fibrosis (IPF), lung cancer (LC), small cell lung carcinoma (SCLC), and lung squamous cell carcinoma (LUSC).

**RESULTS:**

After removing single-nucleotide polymorphisms (SNPs) associated with smoking by the offspring, maternal smoking around birth was associated with increased risk of offspring adult respiratory diseases (OR=1.14; 95% CI: 1.013–1.284; p=0.030), respiratory insufficiency (OR=2.413; 95% CI: 1.039–5.603; p=0.040), COPD (OR=1.14; 95% CI: 1.013–1.284; p=0.003), and asthma (OR=1.336; 95% CI: 1.161–1.538; p<0.001). Besides, maternal smoking during pregnancy was associated with a greater risk of LUSC (OR=1.229; 95% CI: 0.992–1.523; p=0.059) than the risk of IPF (OR=1.001; 95% CI: 0.999–1.003; p=0.224), LC (OR=1.203; 95% CI: 0.964–1.501; p=0.103), or SCLC (OR=1.11; 95% CI: 0.77–1.601; p=0.577).

**CONCLUSIONS:**

In this MR analysis, maternal smoking around birth caused a strong risk factor for the offspring to develop lung problems and CRDs in adulthood. The policy related to smoking cessation for mothers during pregnancy should be encouraged.

## INTRODUCTION

Chronic respiratory diseases (CRDs) accounted for the leading contributor to global mortality in the past decades, seriously endangering human health worldwide. Some of the most common are chronic obstructive pulmonary disease (COPD), asthma, occupational lung diseases, and pulmonary hypertension. Moreover, epidemiological evidence indicated that the incidences of lung cancer (LC) and idiopathic pulmonary fibrosis (IPF) have risen in the past decades, reducing patients’ quality of life.

Smoking is a well-established risk factor for these aforementioned respiratory diseases.

Despite various smoking cessation measures, about 12% of women smoke during pregnancy^1^, resulting in their fetus being exposed to smoke, thus leading to various long-term health problems in the offspring^2,3^. Growing evidence indicates that smoking during pregnancy disturbs fetal lung development^4,5^, causing a negative effect on the pulmonary health of the offspring in childhood with an increased risk for wheezing, hospitalization for respiratory infections, and childhood asthma^6-9^. Whether maternal smoking during pregnancy has a continued impact on the offspring’s lung health during adulthood, remains uncertain. A few previous studies have indicated an association between maternal smoking and adult lung function^10,11^, besides, intrauterine exposure to maternal tobacco smoking was related to more adult respiratory symptoms, but there was no strong evidence that maternal smoking influences adult lung health after multivariable adjustment as these were performed using observational studies, which are vulnerable to confounding bias^12^. A recent study stemming from the UK Biobank cohort reported that maternal smoking might bring about an excess reduction in forced expiratory volume in one second (FEV1)/forced vital capacity (FVC), and risk of COPD, but that the results are heterogeneous due to the individual smoking, and the findings showed that there was no strong evidence that maternal smoking influenced adult lung health among never smokers^13^. Thus, whether maternal smoking around birth represents a strong determinant of CRDs in offspring remains uncertain because the available evidence is scarce.

Undoubtedly, well-designed randomized controlled trials (RCTs) are the gold standard for deducing causality, but their use is frequently limited because of practical and ethical considerations. Mendelian randomization (MR) is a desirable approach that can overcome these challenges by nature, as genetic variants are assorted randomly at conception and fixed at birth; they can be applicable to assess the relationships between maternal smoking and CRDs in their offspring by exploiting genetic variants as instruments for the exposure. Based on data from the largest available genome-wide association study (GWAS), we performed a comprehensive MR study to ascertain the relationships between maternal smoking around birth and a wide range of possible CRDs in their offspring during adulthood.

## METHODS

### Study design

**Figure 1:**
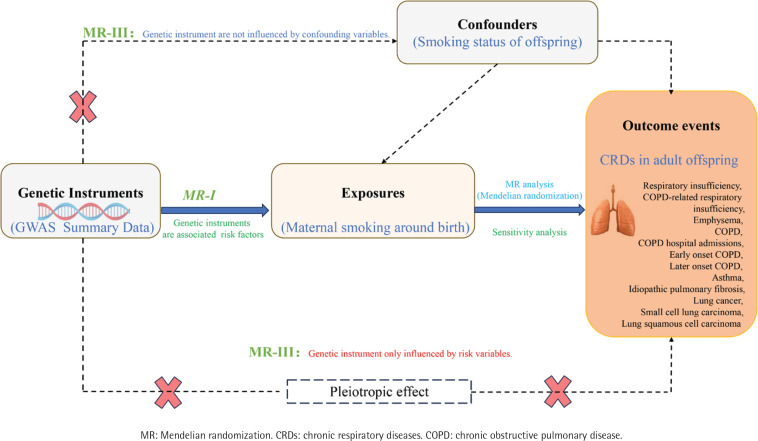
Overall design of the two-sample Mendelian randomization analysis in this study

This is a two-sample MR study design based on summary-level data. An MR analysis depends on the assumptions (Figure 1) that: the genetic variants are strongly associated with the exposure (the relevance assumption); are not associated with confounders of the exposure-outcome relationship (the independence assumption); and have an effect on the outcome through the exposure only and not through any other causal pathway (the exclusion restriction assumption)^14^.

### Data sources and instrumental variable selection

Exposure events were maternal smoking in the time period around birth (as defined in each database), and GWAS data for exposure were obtained from GWAS Catalog: GCST90041844, covering 494132 participants. Outcome events were the CRDs in the offspring during adulthood, including respiratory insufficiency, COPD-related respiratory insufficiency, emphysema, COPD, COPD hospital admissions, early onset COPD, later onset COPD, asthma, idiopathic pulmonary fibrosis (IPF), lung cancer (LC), small cell lung carcinoma (SCLC), and lung squamous cell carcinoma (LUSC). The GWAS data sources for outcomes are described in detail in Table 1.

**Table 1:** The detailed information of GWAS data in outcomes

*Outcomes*	*GWAS ID*	*Sample size*	*Cases*	*Controls*	*p (with SNPs associated with exposure)*
Diseases of the respiratory system	finn-b-J10_RESPIRATORY	218792	107261	111531	5.0×10^-7^
Respiratory insufficiency	finn-b-RESPIRATORYINSUFF	137645	878	136767	5.0×10^-7^
COPD-related respiratory insufficiency	finn-b-COPD_INSUFFICIENCY	187754	1031	186723	5.0×10^-7^
Emphysema	finn-b-J10_EMPHYSEMA	187396	673	186723	5.0×10^-7^
COPD	finn-b-J10_COPD	193638	6915	186723	5.0×10^-7^
COPD, hospital admissions	finn-b-COPD_HOSPITAL	218792	6500	212292	5.0×10^-7^
Early onset COPD	finn-b-COPD_EARLY	215705	3508	212197	5.0×10^-6^
Later onset COPD	finn-b-COPD_LATER	215284	3087	212197	5.0×10^-7^
Asthma	ebi-a-GCST90014325	408422	56167	352255	5.0×10^-7^
Idiopathic pulmonary fibrosis	ebi-a-GCST90018120	437235	1369	435866	5.0×10^-7^
Lung cancer	ieu-a-966	27209	11348	15861	5.0×10^-6^
Small cell lung carcinoma	ieu-a-988	23371	2791	20580	5.0×10^-6^
Squamous cell lung cancer	ieu-a-989	62467	7704	54763	5.0×10^-6^

GWAS: genome-wide association study. COPD: chronic obstructive pulmonary disease. SNPs: single-nucleotide polymorphisms.

As at least 10 instrumental variables (IVs) are required for a MR study^15^, we selected instrumental variables of p<5×10^-7^ or p<5×10^-6^ for MR analysis. The parameters used to eliminate linkage disequilibrium among variables were kb=10000 and r^2^=0.01. The F statistic is used to estimate sample overlap effects and weak instrumental bias, and an F>10 is sufficient to limit bias from weak instrumental variables^16^.

As the smoking status of offspring may affect their risk of developing respiratory diseases, we needed to take this into account in any association, and hence, as the genes rs10226228 were associated with nicotine-dependent smoking of cigarettes per day, and the rs10883802, rs11783093, rs1563245, rs414763, rs414763, rs6011779, rs62477310, and rs7938812 were all related to current tobacco smoking, the rs12042107 and rs876793 were related to past tobacco smoking, while the rs2183947 was related to pack-years of adult smoking as proportion of life span exposed to smoking. Therefore, these single-nucleotide polymorphisms (SNPs) were regarded as an unreliable instrumental variable for maternal smoking around birth (Table 2). Besides, the details of the per allele associations with exposure plotted against per allele associations with outcome are provided in the Supplementary file.

**Table 2:** Detailed information on confounding SNPs that were removed during our GWAS analysis

*Outcomes*	*GWAS ID*	*Removed SNPs related to smoking by offspring*
Diseases of the respiratory system	finn-b-J10_RESPIRATORY	rs10226228, rs10883802, rs11783093, rs12042107, rs2183947, rs576982, rs6011779, rs62477310, rs709400
Respiratory insufficiency	finn-b-RESPIRATORYINSUFF	rs10226228, rs10883802, rs11783093, rs12042107, rs2183947, rs576982, rs6011779, rs62477310, rs709400
COPD-related respiratory insufficiency	finn-b-COPD_INSUFFICIENCY	rs10226228, rs10883802, rs11783093, rs12042107, rs2183947, rs6011779, rs62477310, rs709400
Emphysema	finn-b-J10_EMPHYSEMA	rs10226228, rs10883802, rs11783093, rs12042107, rs2183947, rs576982, rs6011779, rs62477310, rs709400
COPD	finn-b-J10_COPD	rs10226228, rs10883802, rs12042107, rs2183947, rs62477310, rs709400
COPD, hospital admissions	finn-b-COPD_HOSPITAL	rs10226228, rs10883802, rs12042107, rs2183947, rs62477310, rs709400
Early onset COPD	finn-b-COPD_EARLY	rs10226228, rs10883802, rs11783093, rs12042107, rs1563245, rs2183947, rs414763, rs6011779, rs62477310, rs7938812, rs876793
Later onset COPD	finn-b-COPD_LATER	rs10226228, rs10883802, rs11783093, rs12042107, rs2183947, rs576982, rs6011779, rs62477310, rs709400
Asthma	ebi-a-GCST90014325	rs10226228, rs10883802, rs11783093, rs12042107, rs218394, rs6011779, rs62477310, rs709400
Idiopathic pulmonary fibrosis	ebi-a-GCST90018120	rs10226228, rs10883802, rs11783093, rs12042107, rs2183947, rs576982, rs6011779, rs62477310, rs709400
Lung cancer	ieu-a-966	rs10226228, rs10883802, rs2624839, rs414763, rs62477310, rs709400, rs7938812, rs876793
Small cell lung carcinoma	ieu-a-988	rs10226228, rs10883802, rs2624839, rs414763, rs62477310
Squamous cell lung cancer	ieu-a-989	rs10883802, rs26248397, rs414763, rs62477310, rs876793

GWAS: genome-wide association study. COPD: chronic obstructive pulmonary disease. SNPs: single-nucleotide polymorphisms.

### Statistical analysis

We used a two-sample MR analysis to estimate the direct effect of maternal smoking around birth on the risk of offspring CRDs during adulthood . All MR analysis, except for asthma, used fixed-effects models with the inverse-variance-weighted (IVW) model, MR-Egger regression, weighted-median estimator (WME), and weighted mode (VM), while the MR analysis for asthma outcome was conducted using the random effects models. Among these methods, the IVW model is used as the primary method of MR analysis to assess the causal effects, which summarizes effect sizes from multiple independent studies by calculating the weighted mean of the effect sizes using the inverse variance of the individual studies as weights. However, in the presence of horizontal pleiotropy, IVW may not be consistent and may result in the deviation for causal inference. The MR-Egger regression can be used to assess the horizontal pleiotropy of selected IVs, is applied under a weaker assumption that the direct or pleiotropic effects of the genetic variants on the outcome are independent of the genetic associations with the exposure, the so-called ‘instrument strength independent of direct effect’ (InSIDE) assumption^17^. The WME method offers a consistent estimate of causal effects by utilizing the weighted median of Wald under the condition that at least 50% of variants adhere to the criteria of a valid IV for the exclusion restrictions. Utilizing the estimation of individual proportions, the WM method categorizes SNPs based on their similarity and computes the counter-variance weighted count of SNPs in each group^18^.

After removing twelve SNPs (rs10226228, rs10883802, rs11783093, rs1563245, rs414763, rs414763, rs6011779, rs62477310, rs7938812, rs12042107, rs876793, and rs2183947), a leave-one-out sensitivity analysis was performed to examine the effect of individual SNPs on causal estimates. The examination of heterogeneity involved the utilization of Cochran’s Q statistic and the related p-values to ascertain the consistency of causal relationships across all SNPs. The horizontal pleiotropy was calculated based on the MR-Egger intercept and p-values. Besides, MR-Pleiotropy Residual Sum and Outlier (MR-PRESSO) analysis, employed to assess the pleiotropy effects of outlier SNPs and correct abnormal findings attributable to such outliers, involves regressing SNP outcomes on SNP exposure and utilizing the square of residuals to identify outliers.

The sensitivity analyses were conducted by three tests: 1) the leave-one-out sensitivity test was used to determine the stability of individual SNPs in this MR study by excluding IVs in sequence; 2) the robustness of various IVs was tested by Cochrane’s Q-statistic, in which p>0.05 represents non-significant heterogeneity; and 3) the horizontal pleiotropy was calculated based on the MR-Egger intercept and p>0.05 indicates no horizontal pleiotropy.

The results are presented as odds ratios (ORs) with 95% confidence intervals (CIs) for convenience of interpretation. All analyses were performed using R software, version 4.2.0.

## RESULTS

### Results of the Mendelian randomization study testing causal association

The results of MR analysis showed that before removing SNPs related to smoking by the offspring, maternal smoking around birth increased the appearance of respiratory diseases in the offspring by 17% (OR=1.17; 95% CI: 1.05–1.30), increased the risk of respiratory dysfunction by 2.29-fold (OR=3.29; 95% CI: 1.72–6.30), and increased the risk of respiratory dysfunction related to COPD by 3.84-fold (OR=4.84; 95% CI: 2.15–10.91). The risk of developing emphysema increased by 1.85-fold (OR=2.85; 95% CI: 1.12–7.24), the risk of developing COPD increased by 81.6% (OR=1.82; 95% CI: 1.36–2.43), the risk of COPD and hospital admissions increased by 80.3% (OR=1.80; 95% CI: 1.32–2.47), and the risk of early onset COPD increased by 54% (OR=1.54; 95% CI: 1.20–1.972). The risk of developing late onset COPD increased by 66% (OR=1.66; 95% CI: 1.66–4.269), and the risk of developing asthma increased by 24.4% (OR=1.244; 95% CI: 1.11–1.395). The risk of developing IPF increased by 0.2% (OR=1.002; 95% CI: 1.0–1.003), the risk of developing lung cancer increased by 20.4% (OR=1.204; 95% CI: 0.9–1.47), the risk of developing small cell lung cancer increased by 20% (OR=1.20; 95% CI: 0.99–1.47), and the risk of squamous cell lung cancer increased by 24.3% (OR=1.24; 95% CI: 1.02–1.53).

**Figure 2:**
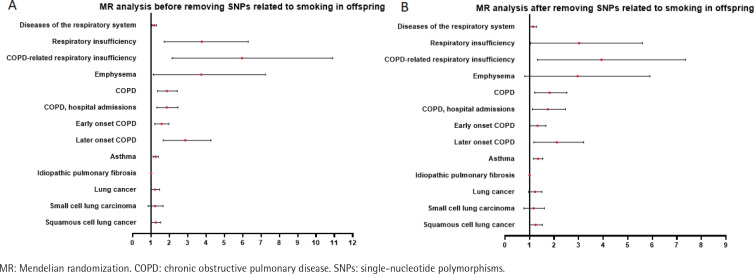
Forest plot of the relationship between maternal smoking before and after birth and chronic respiratory diseases in offspring

After removing SNPs associated with smoking by the offspring, maternal smoking still led to a 14% increase in the risk of respiratory diseases in the offspring (OR=1.14; 95% CI: 1.01–1.28), a 1.41-fold increase in the risk of respiratory insufficiency (OR=2.41; 95% CI: 1.04–5.60), and a 14% increase in the risk of respiratory insufficiency related to COPD (OR=1.14; 95% CI: 1.01–1.28). The risk of COPD increased by 74.2% (OR=1.74; 95% CI: 1.21–2.52), the risk of COPD and hospital admissions increased by 65.9% (OR=1.66; 95% CI: 1.12–2.46), the risk of early onset COPD increased by 29.6% (OR=1.30; 95% CI: 1.01–1.67), the risk of late onset COPD increased by 94.4% (OR=1.95; 95% CI: 1.18–3.21), and the risk of asthma increased by 33.6% (OR=1.336; 95% CI: 1.161–1.538). However, after removing the SNP of smoking by the offspring, the causal relationship between maternal smoking and IPF (OR=1.00; 95% CI: 1.00–1.00), the causal relationship between maternal smoking and lung cancer (OR=1.20; 95% CI: 0.96–1.50), and the causal relationship between maternal smoking and small cell lung cancer (OR=1.11; 95% CI: 0.77–1.60) were no longer statistically significant, while the causal relationship with squamous cell lung cancer (OR=1.23; 95% CI: 0.99–1.52) still existed (Table 3, Figure 2).

**Table 3:** The relationship between maternal smoking and respiratory diseases in the offspring

*Outcome*	*MR analysis before removing SNPs related to smoking by offspring*				*MR analysis after removing SNPs associated with smoking by offspring*			
	*SNPs*	*Methods*	*OR (95% CI)*	*p*	*SNPs*	*Methods*	*OR (95% CI)*	*p*
**Diseases of the respiratory system**	25	Inverse variance weighted	1.171 (1.052–1.303)	0.004	16	Inverse variance weighted	1.14 (1.013–1.284)	0.030
		MR Egger	1.303 (0.806–2.106)	0.292		MR Egger	1.119 (0.654–1.915)	0.687
		Weighted median	1.253 (1.087–1.444)	0.002		Weighted median	1.21 (1.033–1.417)	0.018
		Weighted mode	1.336 (1.007–1.773)	0.056		Weighted mode	1.319 (0.918–1.894)	0.155
**Respiratory insufficiency**	26	Inverse variance weighted	3.292 (1.72–6.298)	<0.001	17	Inverse variance weighted	2.413 (1.039–5.603)	0.040
		MR Egger	68.06 (3.724–1243)	0.009		MR Egger	4.371 (0.088–216.6)	0.470
		Weighted median	2.22 (0.857–5.746)	0.100		Weighted median	1.585 (0.532–4.718)	0.408
		Weighted mode	1.509 (0.201–11.34)	0.693		Weighted mode	1.16 (0.176–7.658)	0.880
**COPD-related respiratory insufficiency**	26	Inverse variance weighted	4.84 (2.148–10.906)	<0.001	18	Inverse variance weighted	3.119 (1.323–7.352)	0.009
		MR Egger	58.99 (1.688–2062)	0.034		MR Egger	4.862 (0.088–268.0)	0.451
		Weighted median	3.233 (1.219–8.58)	0.018		Weighted median	2.187 (0.736–6.501)	0.159
		Weighted mode	2.447 (0.377–15.89)	0.357		Weighted mode	2.168 (0.396–11.86)	0.385
**Emphysema**	26	Inverse variance weighted	2.849 (1.121–7.242)	0.028	17	Inverse variance weighted	2.174 (0.801–5.904)	0.127
		MR Egger	380.8 (8.673–16719)	0.005		MR Egger	63.49 (0.696–5789)	0.092
		Weighted median	2.758 (0.905–8.411)	0.074		Weighted median	1.863 (0.486–7.141)	0.364
		Weighted mode	2.246 (0.269–18.77)	0.462		Weighted mode	1.465 (0.158–13.549)	0.741
**COPD**	23	Inverse variance weighted	1.816 (1.357–2.43)	<0.001	17	Inverse variance weighted	1.742 (1.205–2.519)	0.003
		MR Egger	2.115 (0.514–8.706)	0.311		MR Egger	1.269 (0.217–7.403)	0.795
		Weighted median	1.489 (0.986–2.249)	0.058		Weighted median	1.198 (0.737–1.948)	0.466
		Weighted mode	1.08 (0.469–2.489)	0.858		Weighted mode	1.009 (0.466–2.184)	0.982
**COPD, hospital admissions**	23	Inverse variance weighted	1.803 (1.318–2.467)	<0.001	17	Inverse variance weighted	1.659 (1.119–2.461)	0.012
		MR Egger	1.965 (0.428–9.03)	0.395		MR Egger	1.216 (0.184–8.039)	0.842
		Weighted median	1.512 (1.008–2.267)	0.046		Weighted median	1.316 (0.818–2.115)	0.258
		Weighted mode	1.4 (0.624–3.142)	0.423		Weighted mode	0.992 (0.423–2.325)	0.985
**Early onset COPD**	66	Inverse variance weighted	1.54 (1.204–1.972)	0.001	55	Inverse variance weighted	1.296 (1.009–1.665)	0.043
		MR Egger	0.996 (0.482–2.057)	0.991		MR Egger	0.727 (0.365–1.447)	0.368
		Weighted median	1.428 (1.02–1.998)	0.038		Weighted median	1.295 (0.891–1.88)	0.175
		Weighted mode	1.498 (0.693–3.239)	0.308		Weighted mode	1.333 (0.677–2.623)	0.409
**Later onset COPD**	26	Inverse variance weighted	2.663 (1.661–4.269)	<0.001	17	Inverse variance weighted	1.944 (1.178–3.207)	0.009
		MR Egger	13.821 (1.779–107.374)	0.019		MR Egger	1.658 (0.15–18.266)	0.686
		Weighted median	2.347 (1.314–4.192)	0.004		Weighted median	1.958 (0.996–3.849)	0.051
		Weighted mode	1.828 (0.421–7.934)	0.428		Weighted mode	2.505 (0.642–9.769)	0.205
**Asthma**	26	Inverse variance weighted	1.244 (1.11–1.395)	<0.001	18	Inverse variance weighted	1.336 (1.161–1.538)	<0.001
		MR Egger	0.996 (0.63–1.576)	0.987		MR Egger	0.955 (0.553–1.648)	0.870
		Weighted median	1.176 (1.025–1.349)	0.020		Weighted median	1.187 (1.009–1.397)	0.039
		Weighted mode	1.101 (0.815–1.486)	0.536		Weighted mode	1.139 (0.838–1.55)	0.417
**Idiopathic pulmonary fibrosis**	27	Inverse variance weighted	1.002 (1–1.003)	0.015	18	Inverse variance weighted	1.001 (0.999–1.003)	0.224
		MR Egger	1.003 (0.997–1.009)	0.320		MR Egger	1.002 (0.995–1.01)	0.525
		Weighted median	1.002 (1–1.004)	0.132		Weighted median	1.001 (0.999–1.004)	0.316
		Weighted mode	1.002 (0.998–1.006)	0.271		Weighted mode	1.002 (0.997–1.006)	0.488
**Lung cancer**	45	Inverse variance weighted	1.204 (0.985–1.47)	0.069	37	Inverse variance weighted	1.203 (0.964–1.501)	0.103
		MR Egger	1.168 (0.595–2.296)	0.654		MR Egger	1.288 (0.641–2.591)	0.482
		Weighted median	1.276 (0.98–1.662)	0.071		Weighted median	1.276 (0.948–1.718)	0.108
		Weighted mode	1.339 (0.72–2.489)	0.361		Weighted mode	1.317 (0.671–2.586)	0.429
**Small cell lung carcinoma**	41	Inverse variance weighted	1.179 (0.838–1.657)	0.344	36	Inverse variance weighted	1.11 (0.77–1.601)	0.577
		MR Egger	1.758 (0.391–7.917)	0.467		MR Egger	1.599 (0.334–7.644)	0.560
		Weighted median	1.205 (0.776–1.872)	0.407		Weighted median	1.151 (0.718–1.844)	0.559
		Weighted mode	1.253 (0.494–3.177)	0.637		Weighted mode	1.145 (0.443–2.956)	0.781
**Squamous cell lung cancer**	48	Inverse variance weighted	1.243 (1.015–1.523)	43		Inverse variance weighted	1.229 (0.992–1.523)	0.059
		MR Egger	1.101 (0.557–2.175)			MR Egger	1.144 (0.57–2.295)	0.707
		Weighted median	1.206 (0.904–1.609)			Weighted median	1.205 (0.891–1.631)	0.227
		Weighted mode	1.214 (0.624–2.362)			Weighted mode	1.24 (0.652–2.359)	0.516

COPD: chronic obstructive pulmonary disease. SNPs: single-nucleotide polymorphisms.

### Sensitivity analysis

Our sensitivity analyses included heterogeneity analysis and tests for horizontal pleiotropy (Table 4). After removing confounders associated with offspring smoking, there was no horizontal pleiotropy (p>0.05) in all MR results. Besides, the findings of heterogeneity analysis indicated the absence of statistically significant heterogeneity (p>0.05) in all MR results except for the MR analyses with asthma (Q=30.913, p=0.020) as the outcome event. Moreover, there were no outliers in all MR-PRESSO results.

**Table 4:** Heterogeneity and horizontal pleiotropy in the present Medelian Randomization study

*Outcomes*	*Heterogeneity*		*Horizontal pleiotropy*	
	*Q*	*p*	*Intercept in MR-Egger regression*	*p (MR-Egger intercept analysis)*
Diseases of the respiratory system	12.108	0.671	0.001	0.946
Respiratory insufficiency	10.275	0.852	-0.021	0.764
COPD-related respiratory insufficiency	23.153	0.144	-0.016	0.827
Emphysema	17.134	0.377	-0.120	0.154
COPD	20.910	0.182	0.011	0.723
COPD, hospital admissions	22.759	0.120	0.011	0.746
Early onset COPD	59.961	0.268	0.020	0.084
Later onset COPD	18.228	0.311	0.006	0.896
Asthma	30.913	0.020	0.012	0.230
Idiopathic pulmonary fibrosis	11.727	0.816	0.000	0.728
Lung cancer	17.599	0.996	-0.002	0.840
Small cell lung carcinoma	10.354	1.000	-0.012	0.641
Squamous cell lung cancer	27.813	0.955	0.002	0.833

COPD: chronic obstructive pulmonary disease. SNPs: single-nucleotide polymorphisms.

## DISCUSSION

This study utilized GWAS data to investigate whether the exposure to maternal smoking around birth is associated with CRDs of the offspring during adulthood, as proposed by epidemiologic studies. The results found were: 1) maternal smoking around birth may be defined as a dangerous exposure for lung development in their offspring, inducing respiratory insufficiency, emphysema, and COPD-related respiratory insufficiency; 2) the intrauterine exposure to tobacco smoke may increase the risk of diseases of the respiratory system, especially the chronic airway inflammatory diseases including COPD and asthma; and 3) smoking by pregnant women may result in their offspring being more prone to suffer IPF, and increase the incidence of lung cancer in the offspring, despite that this was not statistically significant.

Tobacco smoke contains thousands of chemical compounds. Nicotine, as one of the leading chemical components in smoke, can enter fetal circulation through the placental barrier and spread throughout the body, which can lead to the development of diseases^19^. In this process, nicotine can interact with nicotinic acetylcholine receptors (nAChRs) in the fetal lung, leading to change in the structure and function of the lung of the offspring^2,20,21^. Smoking in pregnant women has a negative effect on the pulmonary health of their offspring^4^. A prospective study found that FEV1 and forced expiratory flow (FEF) between 25 and 75% of FVC of offspring who had been exposed to maternal smoking *in utero*, and continued to decrease in early adulthood^8^. Meta-analyses have demonstrated a significant association between exposure to maternal smoking during pregnancy and the risk of developing bronchopulmonary dysplasia (BPD)^22^, which might increase the risk of COPD^23^. An animal study reported that maternal exposure to cigarette smoke increased receptors for advanced glycation end-products (RAGE) and in its signaling elements associated with increased oxidative stress and inflammatory cytokines in the offspring’s lungs, inducing the proliferation of lung cells and changing the structure and function of the lung of the offspring, resulting in poor lung function and causing respiratory insufficiency^4^. The limitation of observational studies is that they are susceptible to confounding by unmeasured differences between the exposed and unexposed populations, and our findings provide additional evidence for a potential effect of maternal smoking around birth on their offspring’ poor lung function (including respiratory insufficiency and COPD-related respiratory insufficiency) and pulmonary structural change (such as emphysema).

Cigarette smoking is a key environmental risk factor for chronic airway inflammatory diseases such as asthma and COPD. Previous studies illustrated that maternal smoking poses a risk for their fetus, by altering lung growth and development *in utero*, and possibly priming the immune system by inducing specific epigenetic changes, increasing the morbidity of bronchopulmonary dysplasia (BPD) and leading to COPD in the offspring^24-26^. Our study used SNPs as instrumental variables to elucidate the role of maternal smoking around the time of delivery as a cause of elevated risk of COPD in their offspring. Recently, an MR study has reported that maternal smoking around birth increases the risk of childhood asthma based on childhood asthma of 1993 cases from ukb-d-ASTHMA_CHILD^27^. In contrast, our two-sample MR analysis had a much larger outcome cohort (ebi-a-GCST90014325, including 56167 cases and 352255 controls) and strengthened the evidence for an effect of maternal smoking around birth on their offspring’s asthma during adulthood, providing more convincing evidence by removing SNPs associated with smoking by the offspring in the MR analysis.

Cohort studies have evaluated the longitudinal association of smoking with IPF^28,29^, and an MR study investigated the causal association between smoking and the risk of IPF^30^. Cohort studies have found that smoking could increase the risk of IPF in a dose-response manner, and a two-sample MR study^30,31^ confirmed that a potential causal effect of smoking on IPF, while a one-sample MR study reported that smoking is unlikely to be a causal factor for IPF^31^. Our study found that the offspring might be prone to suffer IPF if they had been exposed to smoking *in utero*, but might be more vulnerable to exposure to tobacco smoking after birth. This study finding strengthens the evidence for an effect of smoking on IPF in people, acquired by exposure^30^.

Smoking has been widely recognized as a risk factor for numerous types of cancer, and studies have confirmed the causal effect of smoking on the risk of various tumors, including lung cancer^32,33^. A clinical study established smoking cessation could decrease the risk of death from lung cancer^34^. In our study, the results indicate that maternal smoking around birth might promote the incidence of lung cancer but could not be defined as a factor for lung cancer owing to the MR analysis after removing SNPs associated with smoking in offspring, providing additional evidence for a causal effect of exposure to smoking after birth on lung cancer.

### Limitations

Although a two-sample MR study is a powerful approach to investigate the relationship between exposures and outcomes, we should be careful with our findings because of several limitations. First, the participants in our study were from the European Pedigree GWAS database. Hence, definitions of exposure to cigarette smoking and its exact timing are defined as categorized in this database. The results, hence, need to be verified in other populations. Second, there may be developmental compensations during offspring growth, which may influence the effects due to instrumental variables. Third, the potential confounding factors, such as the exact timing of maternal smoking around birth and the effects of secondhand smoke on chronic diseases, including CRDs, have not been investigated in this study. Thus, passive smoking may introduce variability in the MR analysis and should be noted to elucidate the effect of maternal smoking around birth on the offspring’s adult lung health and CRDs. Fourth, horizontal pleiotropy is a significant concern for the reliability of MR results. Nevertheless, the MR-Egger regression test showed no clear directional pleiotropy, and the likelihood of this bias is reduced because consistent estimates were observed across multiple MR methods, which have different assumptions.

## CONCLUSIONS

Our study compressively investigated the effect of maternal smoking around birth on the offspring’s adult lung health and CRDs, and the results indicated that smoking during pregnancy may lead to offspring respiratory insufficiency and increase the incidence of chronic airway inflammatory diseases (e.g. asthma and COPD), during adulthood. Thus, it is critical to enhance policies for smoking cessation during pregnancy.

## Supplementary Material



## Data Availability

The data supporting this research are available from the authors on reasonable request.
