# Supplementary material for: The association of maternal smoking around birth with chronic respiratory diseases in adult offspring: A Mendelian randomization study
Source: Tob Induc Dis. 2024 Jun 27;22:10.18332/tid/189394. doi: 10.18332/tid/189394 (PMC11210268; doi:10.18332/tid/189394)
Supplement: Supplementary file 1 [file TID-22-120-s1.pdf]

Table 1 Individual SNP information inclu

exposure	GWAS Catalog	outcome	GWAS ID	SNP	chromosomes	position
maternal smoking around birt	GCST90041844	Diseases of the respiratory system	finn-b-J10_RESPIRATORY	rs1040151	10	126062763
				rs10789442	1	44140075
				rs113331826	11	29861212
				rs113927489	9	136467207
				rs13285789	9	14483853
				rs1569216	4	172473450
				rs34364357	3	140346921
				rs35194198	8	144260893
				rs35566160	2	164928199
				rs36022874	11	113738340
				rs36072649	4	140939110
				rs56322375	17	32906373
				rs62259107	3	67124565
				rs7002049	8	93114414
				rs792458	10	22133793
				rs794375	7	75147801
maternal smoking around birt	GCST90041844	Respiratory insufficiency	finn-b-RESPIRATORYINSUF	rs1040151	10	126062763
				rs10789442	1	44140075
				rs113331826	11	29861212
				rs113927489	9	136467207
				rs13285789	9	14483853
				rs1569216	4	172473450
				rs34364357	3	140346921
				rs35194198	8	144260893
				rs35566160	2	164928199
				rs36022874	11	113738340
				rs36072649	4	140939110
				rs56322375	17	32906373
				rs62259107	3	67124565
				rs6508145	18	50115886

			rs7002049	8	93114414
			rs792458	10	22133793
			rs794375	7	75147801
maternal smoking around birt	GCST90041844	COPD-related respiratory insufficien	finn-b-COPD_INSUFFICIENC	rs1040151	10	126062763
			rs10789442	1	44140075
			rs113331826	11	29861212
			rs113927489	9	136467207
			rs13285789	9	14483853
			rs1569216	4	172473450
			rs34364357	3	140346921
			rs35194198	8	144260893
			rs35566160	2	164928199
			rs36022874	11	113738340
			rs36072649	4	140939110
			rs56322375	17	32906373
			rs576982	15	78870803
			rs62259107	3	67124565
			rs6508145	18	50115886
			rs7002049	8	93114414
			rs792458	10	22133793
			rs794375	7	75147801
maternal smoking around birt	GCST90041844	Emphysema	finn-b-J10_EMPHYSEMA	rs1040151	10	126062763
			rs10789442	1	44140075
			rs113331826	11	29861212
			rs113927489	9	136467207
			rs13285789	9	14483853
			rs1569216	4	172473450
			rs34364357	3	140346921
			rs35194198	8	144260893
			rs35566160	2	164928199
			rs36022874	11	113738340
			rs36072649	4	140939110

				rs56322375	17	32906373
				rs62259107	3	67124565
				rs6508145	18	50115886
				rs7002049	8	93114414
				rs792458	10	22133793
				rs794375	7	75147801
maternal smoking around birt	GCST90041844	COPD	finn-b-J10_COPD	rs1040151	10	126062763
				rs10789442	1	44140075
				rs113331826	11	29861212
				rs113927489	9	136467207
				rs13285789	9	14483853
				rs1569216	4	172473450
				rs34364357	3	140346921
				rs35194198	8	144260893
				rs35566160	2	164928199
				rs36022874	11	113738340
				rs36072649	4	140939110
				rs56322375	17	32906373
				rs62259107	3	67124565
				rs6508145	18	50115886
				rs7002049	8	93114414
				rs792458	10	22133793
				rs794375	7	75147801
maternal smoking around birt	GCST90041844	COPD, hospital admissions	finn-b-COPD_HOSPITAL	rs1040151	10	126062763
				rs10789442	1	44140075
				rs113331826	11	29861212
				rs113927489	9	136467207
				rs13285789	9	14483853
				rs1569216	4	172473450
				rs34364357	3	140346921
				rs35194198	8	144260893
				rs35566160	2	164928199

				rs36022874	11	113738340
				rs36072649	4	140939110
				rs56322375	17	32906373
				rs62259107	3	67124565
				rs6508145	18	50115886
				rs7002049	8	93114414
				rs792458	10	22133793
				rs794375	7	75147801
maternal smoking around birt	GCST90041844	Early onset COPD	finn-b-COPD_EARLY	rs1040151	10	126062763
				rs10789442	1	44140075
				rs111860035	3	5021325
				rs11210247	1	73900932
				rs113331826	11	29861212
				rs113927489	9	136467207
				rs114000606	1	11333812
				rs11653367	17	44191483
				rs13123903	4	35513601
				rs13285789	9	14483853
				rs13356444	5	30919960
				rs1371274	18	1837007
				rs138425427	16	30591862
				rs1438299	5	12925979
				rs1569216	4	172473450
				rs17133347	7	3472721
				rs1958671	14	57363484
				rs254945	16	8956703
				rs2624839	3	50202231
				rs2693286	2	199703219
				rs2710634	2	32808804
				rs322640	18	34592625
				rs34364357	3	140346921
				rs34832954	10	107364513

				rs35194198	8	144260893
				rs35566160	2	164928199
				rs36022874	11	113738340
				rs36072649	4	140939110
				rs3796432	4	96030402
				rs38849	7	116332539
				rs4551449	9	22610366
				rs4557199	3	77069266
				rs4654327	1	29190138
				rs4757365	11	15815555
				rs4865667	5	50748173
				rs517680	1	112356899
				rs55741516	14	42697579
				rs56322375	17	32906373
				rs575030	11	121092398
				rs5762858	22	29249002
				rs6120777	20	33560172
				rs62259107	3	67124565
				rs6508145	18	50115886
				rs6706169	2	166281132
				rs676217	11	30195757
				rs6985180	8	115759747
				rs7002049	8	93114414
				rs709400	14	104149475
				rs73430670	11	27615630
				rs7766061	6	131967033
				rs7770593	6	24310319
				rs78420531	17	43249246
				rs792458	10	22133793
				rs794375	7	75147801
				rs9375772	6	131143651
maternal smoking around birt	GCST90041844	Later onset COPD	finn-b-COPD_LATER	rs1040151	10	126062763

				rs10789442	1	44140075
				rs113331826	11	29861212
				rs113927489	9	136467207
				rs13285789	9	14483853
				rs1569216	4	172473450
				rs34364357	3	140346921
				rs35194198	8	144260893
				rs35566160	2	164928199
				rs36022874	11	113738340
				rs36072649	4	140939110
				rs56322375	17	32906373
				rs62259107	3	67124565
				rs6508145	18	50115886
				rs7002049	8	93114414
				rs792458	10	22133793
				rs794375	7	75147801
maternal smoking around birt	GCST90041844	Asthma	ebi-a-GCST90014325	rs1040151	10	126062763
				rs10789442	1	44140075
				rs113331826	11	29861212
				rs113927489	9	136467207
				rs12920941	16	24830866
				rs13285789	9	14483853
				rs1569216	4	172473450
				rs34364357	3	140346921
				rs35194198	8	144260893
				rs35566160	2	164928199
				rs36022874	11	113738340
				rs36072649	4	140939110
				rs56322375	17	32906373
				rs62259107	3	67124565
				rs6508145	18	50115886
				rs7002049	8	93114414

			rs792458	10	22133793
			rs794375	7	75147801
maternal smoking around birt GCST90041844	Idiopathic pulmonary fibrosis	ebi-a-GCST90018120	rs1040151	10	126062763
			rs10789442	1	44140075
			rs113331826	11	29861212
			rs113927489	9	136467207
			rs12920941	16	24830866
			rs13285789	9	14483853
			rs1569216	4	172473450
			rs34364357	3	140346921
			rs35194198	8	144260893
			rs35566160	2	164928199
			rs36022874	11	113738340
			rs36072649	4	140939110
			rs56322375	17	32906373
			rs62259107	3	67124565
			rs6508145	18	50115886
			rs7002049	8	93114414
			rs792458	10	22133793
			rs794375	7	75147801
maternal smoking around birt GCST90041844	Lung cancer	ieu-a-966	rs111860035	3	5021325
			rs113331826	11	29861212
			rs113927489	9	136467207
			rs13285789	9	14483853
			rs13356444	5	30919960
			rs1371274	18	1837007
			rs138425427	16	30591862
			rs1438299	5	12925979
			rs1569216	4	172473450
			rs17133347	7	3472721
			rs1958671	14	57363484
			rs254945	16	8956703

				rs2693286	2	199703219
				rs322640	18	34592625
				rs34832954	10	107364513
				rs35194198	8	144260893
				rs35566160	2	164928199
				rs36022874	11	113738340
				rs3796432	4	96030402
				rs38849	7	116332539
				rs4551449	9	22610366
				rs4654327	1	29190138
				rs4757365	11	15815555
				rs4865667	5	50748173
				rs517680	1	112356899
				rs55741516	14	42697579
				rs56322375	17	32906373
				rs6508145	18	50115886
				rs6706169	2	166281132
				rs676217	11	30195757
				rs6985180	8	115759747
				rs7002049	8	93114414
				rs7766061	6	131967033
				rs78420531	17	43249246
				rs792458	10	22133793
				rs794375	7	75147801
				rs9375772	6	131143651
maternal smoking around birt	GCST90041844	Small cell lung carcinoma	ieu-a-988	rs1040151	10	126062763
				rs10789442	1	44140075
				rs11210247	1	73900932
				rs113331826	11	29861212
				rs12920941	16	24830866
				rs13356444	5	30919960
				rs1371274	18	1837007

				rs1438299	5	12925979
				rs1569216	4	172473450
				rs254945	16	8956703
				rs34364357	3	140346921
				rs34832954	10	107364513
				rs35194198	8	144260893
				rs36022874	11	113738340
				rs36072649	4	140939110
				rs3796432	4	96030402
				rs41860	7	69001742
				rs4551449	9	22610366
				rs4557199	3	77069266
				rs4654327	1	29190138
				rs4757365	11	15815555
				rs4865667	5	50748173
				rs517680	1	112356899
				rs56322375	17	32906373
				rs575030	11	121092398
				rs5762858	22	29249002
				rs6120777	20	33560172
				rs6508145	18	50115886
				rs676217	11	30195757
				rs7002049	8	93114414
				rs73430670	11	27615630
				rs7766061	6	131967033
				rs7770593	6	24310319
				rs78420531	17	43249246
				rs794375	7	75147801
				rs9375772	6	131143651
maternal smoking around birt	GCST90041844	Squamous cell lung cancer	ieu-a-989	rs1040151	10	126062763
				rs111860035	3	5021325
				rs11210247	1	73900932

rs113331826	11	29861212
rs12920941	16	24830866
rs13285789	9	14483853
rs13356444	5	30919960
rs1371274	18	1837007
rs138425427	16	30591862
rs1438299	5	12925979
rs1569216	4	172473450
rs1958671	14	57363484
rs254945	16	8956703
rs2693286	2	199703219
rs2710634	2	32808804
rs322640	18	34592625
rs34364357	3	140346921
rs34832954	10	107364513
rs35194198	8	144260893
rs36022874	11	113738340
rs36072649	4	140939110
rs3796432	4	96030402
rs41860	7	69001742
rs4551449	9	22610366
rs4654327	1	29190138
rs4757365	11	15815555
rs4865667	5	50748173
rs517680	1	112356899
rs55741516	14	42697579
rs56322375	17	32906373
rs575030	11	121092398
rs6120777	20	33560172
rs62259107	3	67124565
rs6508145	18	50115886
rs6706169	2	166281132

rs676217	11	30195757
rs6985180	8	115759747
rs7002049	8	93114414
rs73430670	11	27615630
rs7766061	6	131967033
rs78420531	17	43249246
rs794375	7	75147801
rs9375772	6	131143651

uded in Mendelian randomization analysis

effect_allele.exposure	other_allele.exposure	effect_allele.outcome	other_allele.outcom	beta.exposure	beta.outcome	eaf.exposure
G	A	G	A	0,0360543	-0,0042	0,186186
C	A	C	A	-0,036768	0,0063	0,333704
C	T	C	T	-0,0844322	-0,008	0,0242447
G	C	G	C	0,058657	0,0254	0,110208
G	A	G	A	0,0409708	0,0066	0,116584
A	G	A	G	-0,028419	-0,0064	0,342582
T	A	T	A	-0,0769611	0,0123	0,0320261
G	A	G	A	-0,0279554	0,0009	0,334699
G	A	G	A	0,0300218	0,0084	0,270353
C	T	C	T	0,0333111	0,0124	0,209524
A	T	A	T	-0,0348985	-0,0108	0,381357
T	G	T	G	0,0347809	0,0139	0,230467
G	C	G	C	0,0459493	0,0036	0,0933564
T	C	T	C	-0,038054	-0,0086	0,214316
T	G	T	G	0,0281629	0,009	0,316275
C	T	C	T	-0,0272062	0,0052	0,425782
G	A	G	A	0,0360543	0,0482	0,186186
C	A	C	A	-0,036768	-0,0909	0,333704
C	T	C	T	-0,0844322	0,0899	0,0242447
G	C	G	C	0,058657	0,26	0,110208
G	A	G	A	0,0409708	0,0022	0,116584
A	G	A	G	-0,028419	0,0018	0,342582
T	A	T	A	-0,0769611	0,0499	0,0320261
G	A	G	A	-0,0279554	0,0048	0,334699
G	A	G	A	0,0300218	-0,0131	0,270353
C	T	C	T	0,0333111	0,0099	0,209524
A	T	A	T	-0,0348985	-0,035	0,381357
T	G	T	G	0,0347809	0,0182	0,230467
G	C	G	C	0,0459493	0,0273	0,0933564
A	G	A	G	-0,0352976	-0,1438	0,191345

T	C	T	C	-0,038054	-0,0057	0,214316
T	G	T	G	0,0281629	0,0227	0,316275
C	T	C	T	-0,0272062	0,0007	0,425782
G	A	G	A	0,0360543	0,0553	0,186186
C	A	C	A	-0,036768	-0,0182	0,333704
C	T	C	T	-0,0844322	0,0718	0,0242447
G	C	G	C	0,058657	0,2202	0,110208
G	A	G	A	0,0409708	0,0223	0,116584
A	G	A	G	-0,028419	-0,0216	0,342582
T	A	T	A	-0,0769611	0,2045	0,0320261
G	A	G	A	-0,0279554	0,0426	0,334699
G	A	G	A	0,0300218	0,0741	0,270353
C	T	C	T	0,0333111	-0,0118	0,209524
A	T	A	T	-0,0348985	-0,0471	0,381357
T	G	T	G	0,0347809	0,0308	0,230467
T	C	T	C	-0,0453606	-0,1839	0,227239
G	C	G	C	0,0459493	-0,103	0,0933564
A	G	A	G	-0,0352976	-0,1423	0,191345
T	C	T	C	-0,038054	-0,0174	0,214316
T	G	T	G	0,0281629	0,0439	0,316275
C	T	C	T	-0,0272062	0,0062	0,425782
G	A	G	A	0,0360543	-0,0463	0,186186
C	A	C	A	-0,036768	-0,0813	0,333704
C	T	C	T	-0,0844322	-0,3168	0,0242447
G	C	G	C	0,058657	0,1153	0,110208
G	A	G	A	0,0409708	0,0815	0,116584
A	G	A	G	-0,028419	-0,0179	0,342582
T	A	T	A	-0,0769611	-0,2798	0,0320261
G	A	G	A	-0,0279554	-0,1285	0,334699
G	A	G	A	0,0300218	-0,0212	0,270353
C	T	C	T	0,0333111	0,0184	0,209524
A	T	A	T	-0,0348985	0,0113	0,381357

T	G	T	G	0,0347809	-0,0281	0,230467
G	C	G	C	0,0459493	0,0332	0,0933564
A	G	A	G	-0,0352976	-0,007	0,191345
T	C	T	C	-0,038054	-0,0472	0,214316
T	G	T	G	0,0281629	0,0945	0,316275
C	T	C	T	-0,0272062	0,1132	0,425782
G	A	G	A	0,0360543	-0,0024	0,186186
C	A	C	A	-0,036768	0,0078	0,333704
C	T	C	T	-0,0844322	-0,0529	0,0242447
G	C	G	C	0,058657	0,1338	0,110208
G	A	G	A	0,0409708	0,0064	0,116584
A	G	A	G	-0,028419	-0,0268	0,342582
T	A	T	A	-0,0769611	0,0414	0,0320261
G	A	G	A	-0,0279554	-0,0042	0,334699
G	A	G	A	0,0300218	0,0267	0,270353
C	T	C	T	0,0333111	0,0081	0,209524
A	T	A	T	-0,0348985	-0,0527	0,381357
T	G	T	G	0,0347809	0,0443	0,230467
G	C	G	C	0,0459493	-0,0222	0,0933564
A	G	A	G	-0,0352976	-0,0562	0,191345
T	C	T	C	-0,038054	0,002	0,214316
T	G	T	G	0,0281629	0,04	0,316275
C	T	C	T	-0,0272062	0,0004	0,425782
G	A	G	A	0,0360543	0,0074	0,186186
C	A	C	A	-0,036768	0,0121	0,333704
C	T	C	T	-0,0844322	-0,0355	0,0242447
G	C	G	C	0,058657	0,1581	0,110208
G	A	G	A	0,0409708	-0,0002	0,116584
A	G	A	G	-0,028419	-0,0304	0,342582
T	A	T	A	-0,0769611	0,0383	0,0320261
G	A	G	A	-0,0279554	0,0069	0,334699
G	A	G	A	0,0300218	0,0269	0,270353

C	T	C	T	0,0333111	0,003	0,209524
A	T	A	T	-0,0348985	-0,0568	0,381357
T	G	T	G	0,0347809	0,0386	0,230467
G	C	G	C	0,0459493	-0,0275	0,0933564
A	G	A	G	-0,0352976	-0,0505	0,191345
T	C	T	C	-0,038054	0,0144	0,214316
T	G	T	G	0,0281629	0,0279	0,316275
C	T	C	T	-0,0272062	-0,0102	0,425782
G	A	G	A	0,0360543	-0,0286	0,186186
C	A	C	A	-0,036768	-0,0046	0,333704
G	A	G	A	-0,208069	0,098	0,00325584
T	C	T	C	0,0243646	-0,0196	0,498365
C	T	C	T	-0,0844322	-0,0294	0,0242447
G	C	G	C	0,058657	0,1255	0,110208
T	C	T	C	0,106482	-0,2818	0,0130424
G	A	G	A	-0,0252294	-0,0174	0,326882
T	C	T	C	0,0238921	0,0402	0,494703
G	A	G	A	0,0409708	0,0152	0,116584
C	T	C	T	-0,0251824	-0,0299	0,403951
C	A	C	A	0,0373282	0,0301	0,136786
G	C	G	C	0,0377768	-0,0042	0,112165
T	C	T	C	0,0241589	-0,016	0,426729
A	G	A	G	-0,028419	-0,0434	0,342582
T	C	T	C	-0,0426493	0,023	0,100259
G	A	G	A	0,024792	0,0169	0,480478
G	A	G	A	0,0277688	0,0054	0,264158
C	T	C	T	0,0259197	0,0251	0,427996
C	T	C	T	0,0247023	-0,0089	0,449018
T	C	T	C	0,0258716	0,0137	0,477374
T	A	T	A	-0,0423234	-0,0811	0,0926798
T	A	T	A	-0,0769611	0,1395	0,0320261
T	C	T	C	-0,0560452	-0,0411	0,0500616

G	A	G	A	-0,0279554	0,0566	0,334699
G	A	G	A	0,0300218	0,051	0,270353
C	T	C	T	0,0333111	-0,0085	0,209524
A	T	A	T	-0,0348985	-0,0692	0,381357
T	G	T	G	-0,0256758	-0,0701	0,365529
C	G	C	G	0,0295997	0,0104	0,251969
C	T	C	T	0,0351041	-0,0306	0,148641
C	T	C	T	-0,0359164	-0,0047	0,132981
G	A	G	A	0,0242298	0,025	0,483134
T	C	T	C	0,0260285	-0,029	0,293209
T	C	T	C	-0,0260678	-0,0282	0,388073
A	G	A	G	0,0364123	-0,0196	0,142365
T	C	T	C	0,0253588	0,0067	0,360429
T	G	T	G	0,0347809	0,0436	0,230467
C	T	C	T	0,0266244	-0,0071	0,336365
G	A	G	A	0,0308548	-0,0072	0,17613
A	G	A	G	-0,0330705	0,0005	0,157681
G	C	G	C	0,0459493	-0,0535	0,0933564
A	G	A	G	-0,0352976	-0,0206	0,191345
A	G	A	G	-0,0283244	0,0061	0,234961
G	T	G	T	0,0247884	-0,0343	0,364437
A	G	A	G	0,023868	0,0097	0,473614
T	C	T	C	-0,038054	-0,0054	0,214316
G	A	G	A	-0,0267268	-0,0274	0,373664
G	A	G	A	0,0285864	0,0619	0,237227
A	G	A	G	0,0310527	0,0008	0,178585
A	G	A	G	0,0255081	0,008	0,325889
T	G	T	G	0,0596848	0,0213	0,0409855
T	G	T	G	0,0281629	0,0179	0,316275
C	T	C	T	-0,0272062	-0,0112	0,425782
T	C	T	C	0,0366565	0,0303	0,140892
G	A	G	A	0,0360543	0,0432	0,186186

C	A	C	A	-0,036768	0,0318	0,333704
C	T	C	T	-0,0844322	-0,0474	0,0242447
G	C	G	C	0,058657	0,1492	0,110208
G	A	G	A	0,0409708	-0,0135	0,116584
A	G	A	G	-0,028419	-0,0218	0,342582
T	A	T	A	-0,0769611	-0,09	0,0320261
G	A	G	A	-0,0279554	-0,0548	0,334699
G	A	G	A	0,0300218	-0,0125	0,270353
C	T	C	T	0,0333111	0,0196	0,209524
A	T	A	T	-0,0348985	-0,0438	0,381357
T	G	T	G	0,0347809	0,032	0,230467
G	C	G	C	0,0459493	0,0114	0,0933564
A	G	A	G	-0,0352976	-0,0875	0,191345
T	C	T	C	-0,038054	0,0287	0,214316
T	G	T	G	0,0281629	0,0477	0,316275
C	T	C	T	-0,0272062	-0,0041	0,425782
G	A	G	A	0,0360543	0,0105715	0,186186
C	A	C	A	-0,036768	-0,00630682	0,333704
C	T	C	T	-0,0844322	-0,0109507	0,0242447
G	C	G	C	0,058657	0,0230071	0,110208
A	T	A	T	-0,0322529	-0,00553128	0,273445
G	A	G	A	0,0409708	-0,000648317	0,116584
A	G	A	G	-0,028419	-0,0136593	0,342582
T	A	T	A	-0,0769611	0,000489299	0,0320261
G	A	G	A	-0,0279554	0,00514858	0,334699
G	A	G	A	0,0300218	0,00479296	0,270353
C	T	C	T	0,0333111	0,0252733	0,209524
A	T	A	T	-0,0348985	-0,023216	0,381357
T	G	T	G	0,0347809	-0,00512607	0,230467
G	C	G	C	0,0459493	0,000956817	0,0933564
A	G	A	G	-0,0352976	-0,00398298	0,191345
T	C	T	C	-0,038054	-0,018291	0,214316

T	G	T	G	0,0281629	0,0197405	0,316275
C	T	C	T	-0,0272062	-0,022725	0,425782
G	A	G	A	0,0360543	-0,000153066	0,186186
C	A	C	A	-0,036768	-4,49087E-05	0,333704
C	T	C	T	-0,0844322	-0,000580345	0,0242447
G	C	G	C	0,058657	7,35751E-05	0,110208
A	T	A	T	-0,0322529	-9,36976E-05	0,273445
G	A	G	A	0,0409708	-0,000139297	0,116584
A	G	A	G	-0,028419	0,000139731	0,342582
T	A	T	A	-0,0769611	0,00008952	0,0320261
G	A	G	A	-0,0279554	-7,17822E-05	0,334699
G	A	G	A	0,0300218	3,91133E-05	0,270353
C	T	C	T	0,0333111	0,000271529	0,209524
A	T	A	T	-0,0348985	-5,73318E-05	0,381357
T	G	T	G	0,0347809	0,000145894	0,230467
G	C	G	C	0,0459493	0,000179162	0,0933564
A	G	A	G	-0,0352976	0,000032598	0,191345
T	C	T	C	-0,038054	5,81648E-05	0,214316
T	G	T	G	0,0281629	0,000084654	0,316275
C	T	C	T	-0,0272062	-1,06266E-05	0,425782
G	A	G	A	-0,208069	0,115374	0,00325584
C	T	C	T	-0,0844322	-0,0536951	0,0242447
G	C	G	C	0,058657	0,0612437	0,110208
G	A	G	A	0,0409708	-0,0288533	0,116584
C	T	C	T	-0,0251824	-0,00842338	0,403951
C	A	C	A	0,0373282	-0,00743356	0,136786
G	C	G	C	0,0377768	0,0161558	0,112165
T	C	T	C	0,0241589	-0,0166498	0,426729
A	G	A	G	-0,028419	-0,0149055	0,342582
T	C	T	C	-0,0426493	-0,00630987	0,100259
G	A	G	A	0,024792	0,013463	0,480478
G	A	G	A	0,0277688	-0,00612271	0,264158

C	T	C	T	0,0247023	-0,000237028	0,449018
T	A	T	A	-0,0423234	-0,0339891	0,0926798
T	C	T	C	-0,0560452	-0,0282462	0,0500616
G	A	G	A	-0,0279554	-0,0106343	0,334699
G	A	G	A	0,0300218	0,00546106	0,270353
C	T	C	T	0,0333111	0,028397	0,209524
T	G	T	G	-0,0256758	0,00158774	0,365529
C	G	C	G	0,0295997	0,0111575	0,251969
C	T	C	T	0,0351041	0,000535856	0,148641
G	A	G	A	0,0242298	0,00590453	0,483134
T	C	T	C	0,0260285	-0,00181665	0,293209
T	C	T	C	-0,0260678	0,00129017	0,388073
A	G	A	G	0,0364123	-0,017139	0,142365
T	C	T	C	0,0253588	-0,0217161	0,360429
T	G	T	G	0,0347809	0,00878827	0,230467
A	G	A	G	-0,0352976	-0,0277803	0,191345
A	G	A	G	-0,0283244	-0,0148385	0,234961
G	T	G	T	0,0247884	-0,00121974	0,364437
A	G	A	G	0,023868	0,00750874	0,473614
T	C	T	C	-0,038054	-0,00957671	0,214316
A	G	A	G	0,0310527	0,0259952	0,178585
T	G	T	G	0,0596848	0,0318378	0,0409855
T	G	T	G	0,0281629	0,00535364	0,316275
C	T	C	T	-0,0272062	0,00428182	0,425782
T	C	T	C	0,0366565	0,00693688	0,140892
G	A	G	A	0,0360543	0,0191711	0,186186
C	A	C	A	-0,036768	-0,0238153	0,333704
T	C	T	C	0,0243646	-0,0210597	0,498365
C	T	C	T	-0,0844322	-0,0940217	0,0242447
A	T	A	T	-0,0322529	-0,0373551	0,273445
C	T	C	T	-0,0251824	-0,00201503	0,403951
C	A	C	A	0,0373282	0,0105749	0,136786

T	C	T	C	0,0241589	0,0123563	0,426729
A	G	A	G	-0,028419	0,0231636	0,342582
G	A	G	A	0,0277688	-0,0020962	0,264158
T	A	T	A	-0,0769611	0,0847951	0,0320261
T	C	T	C	-0,0560452	-0,0359008	0,0500616
G	A	G	A	-0,0279554	0,0213367	0,334699
C	T	C	T	0,0333111	0,038912	0,209524
A	T	A	T	-0,0348985	0,00971565	0,381357
T	G	T	G	-0,0256758	-0,0204231	0,365529
T	A	T	A	-0,025676	-0,0204701	0,361487
C	T	C	T	0,0351041	0,0213906	0,148641
C	T	C	T	-0,0359164	-0,00502561	0,132981
G	A	G	A	0,0242298	-0,00907304	0,483134
T	C	T	C	0,0260285	-0,000933436	0,293209
T	C	T	C	-0,0260678	-0,00529399	0,388073
A	G	A	G	0,0364123	0,0273457	0,142365
T	G	T	G	0,0347809	-0,0194275	0,230467
C	T	C	T	0,0266244	-0,0228705	0,336365
G	A	G	A	0,0308548	-0,00058317	0,17613
A	G	A	G	-0,0330705	0,0228079	0,157681
A	G	A	G	-0,0352976	0,00720796	0,191345
G	T	G	T	0,0247884	-0,00262344	0,364437
T	C	T	C	-0,038054	-0,0115009	0,214316
G	A	G	A	0,0285864	0,00859991	0,237227
A	G	A	G	0,0310527	-0,00506983	0,178585
A	G	A	G	0,0255081	0,0036852	0,325889
T	G	T	G	0,0596848	0,0110102	0,0409855
C	T	C	T	-0,0272062	-0,00284304	0,425782
T	C	T	C	0,0366565	-0,0145392	0,140892
G	A	G	A	0,0360543	0,0218036	0,186186
G	A	G	A	-0,208069	-0,0116091	0,00325584
T	C	T	C	0,0243646	0,0144631	0,498365

C	T	C	T	-0,0844322	-0,0292095	0,0242447
A	T	A	T	-0,0322529	-0,00804729	0,273445
G	A	G	A	0,0409708	-0,0287318	0,116584
C	T	C	T	-0,0251824	-0,0167261	0,403951
C	A	C	A	0,0373282	-0,00673563	0,136786
G	C	G	C	0,0377768	-0,00120973	0,112165
T	C	T	C	0,0241589	-0,00525076	0,426729
A	G	A	G	-0,028419	-0,00705483	0,342582
G	A	G	A	0,024792	0,0248663	0,480478
G	A	G	A	0,0277688	-0,000388075	0,264158
C	T	C	T	0,0247023	-0,0181792	0,449018
T	C	T	C	0,0258716	0,0316023	0,477374
T	A	T	A	-0,0423234	-0,0229514	0,0926798
T	A	T	A	-0,0769611	-0,0141304	0,0320261
T	C	T	C	-0,0560452	-0,0460942	0,0500616
G	A	G	A	-0,0279554	-0,0211971	0,334699
C	T	C	T	0,0333111	0,0297355	0,209524
A	T	A	T	-0,0348985	-0,0440794	0,381357
T	G	T	G	-0,0256758	6,49979E-05	0,365529
T	A	T	A	-0,025676	-0,0133568	0,361487
C	T	C	T	0,0351041	0,00482633	0,148641
G	A	G	A	0,0242298	-0,0177771	0,483134
T	C	T	C	0,0260285	-0,0127996	0,293209
T	C	T	C	-0,0260678	-0,0287287	0,388073
A	G	A	G	0,0364123	-0,00470103	0,142365
T	C	T	C	0,0253588	-0,0227488	0,360429
T	G	T	G	0,0347809	0,0142904	0,230467
C	T	C	T	0,0266244	0,00498754	0,336365
A	G	A	G	-0,0330705	-0,0315165	0,157681
G	C	G	C	0,0459493	-0,0184053	0,0933564
A	G	A	G	-0,0352976	-0,018713	0,191345
A	G	A	G	-0,0283244	-0,00839413	0,234961

G	T	G	T	0,0247884	-0,0157209	0,364437
A	G	A	G	0,023868	0,0103146	0,473614
T	C	T	C	-0,038054	0,0202339	0,214316
G	A	G	A	0,0285864	0,00381471	0,237227
A	G	A	G	0,0310527	-0,0154172	0,178585
T	G	T	G	0,0596848	-0,031405	0,0409855
C	T	C	T	-0,0272062	0,00238116	0,425782
T	C	T	C	0,0366565	0,0251481	0,140892

eaf.outcome	se.exposure	se.outcome	pval.exposure	pval.outcome	F
0,3279	0,0067914	0,0071	1,10343E-07	0,554201	145,65
0,3569	0,00547216	0,0069	1,8284E-11	0,3655	233,71
0,01957	0,0167047	0,024	4,31733E-07	0,739301	131,48
0,03256	0,00823623	0,0188	1,06507E-12	0,1764	261,65
0,1482	0,00802224	0,0093	3,27047E-07	0,4778	134,55
0,3133	0,00549191	0,0072	2,28278E-07	0,3712	138,34
0,01288	0,0147306	0,0296	1,74561E-07	0,6783	141,29
0,2604	0,00544061	0,0075	2,77262E-07	0,9068	136,25
0,2558	0,00591995	0,0076	3,95134E-07	0,2703	133,07
0,2251	0,00630708	0,0079	1,28103E-07	0,1177	143,67
0,3625	0,00530438	0,0069	4,73059E-11	0,1163	223,84
0,2046	0,00629715	0,0083	3,32745E-08	0,09378	157,19
0,06698	0,00885138	0,0133	2,08938E-07	0,784401	139,08
0,1838	0,00628848	0,0085	1,43603E-09	0,3118	188,88
0,3079	0,00553787	0,0072	3,66622E-07	0,2077	133,58
0,5142	0,00523984	0,0067	2,07845E-07	0,4327	139,29
0,3286	0,0067914	0,0521	1,10343E-07	0,3552	145,65
0,3565	0,00547216	0,0508	1,8284E-11	0,0735005	233,71
0,01971	0,0167047	0,173	4,31733E-07	0,6033	131,48
0,0326	0,00823623	0,1367	1,06507E-12	0,0572005	261,65
0,1481	0,00802224	0,0679	3,27047E-07	0,9743	134,55
0,314	0,00549191	0,0523	2,28278E-07	0,9728	138,34
0,01289	0,0147306	0,2174	1,74561E-07	0,8183	141,29
0,2598	0,00544061	0,0553	2,77262E-07	0,9315	136,25
0,2552	0,00591995	0,056	3,95134E-07	0,8148	133,07
0,2246	0,00630708	0,0582	1,28103E-07	0,8654	143,67
0,3643	0,00530438	0,0504	4,73059E-11	0,487	223,84
0,2029	0,00629715	0,0607	3,32745E-08	0,764401	157,19
0,06681	0,00885138	0,0973	2,08938E-07	0,779399	139,08
0,1938	0,00650188	0,0611	5,67258E-08	0,0186599	150,87

0,1844	0,00628848	0,0626	1,43603E-09	0,9268	188,88
0,307	0,00553787	0,0525	3,66622E-07	0,6655	133,58
0,5137	0,00523984	0,0487	2,07845E-07	0,9884	139,29
0,3278	0,0067914	0,0485	1,10343E-07	0,2544	145,65
0,3569	0,00547216	0,0471	1,8284E-11	0,6995	233,71
0,01961	0,0167047	0,1611	4,31733E-07	0,655901	131,48
0,03248	0,00823623	0,1252	1,06507E-12	0,0787209	261,65
0,148	0,00802224	0,0636	3,27047E-07	0,7264	134,55
0,3137	0,00549191	0,0486	2,28278E-07	0,6566	138,34
0,013	0,0147306	0,1936	1,74561E-07	0,2908	141,29
0,2608	0,00544061	0,0513	2,77262E-07	0,406	136,25
0,2556	0,00591995	0,0519	3,95134E-07	0,1533	133,07
0,2244	0,00630708	0,0544	1,28103E-07	0,8287	143,67
0,3632	0,00530438	0,0468	4,73059E-11	0,3146	223,84
0,2043	0,00629715	0,056	3,32745E-08	0,5825	157,19
0,2869	0,0061354	0,0499	1,4327E-13	0,0002262	283,46
0,06689	0,00885138	0,0899	2,08938E-07	0,2521	139,08
0,193	0,00650188	0,0568	5,67258E-08	0,01217	150,87
0,1841	0,00628848	0,0582	1,43603E-09	0,764401	188,88
0,3069	0,00553787	0,0488	3,66622E-07	0,3689	133,58
0,5143	0,00523984	0,0453	2,07845E-07	0,891	139,29
0,3277	0,0067914	0,0596	1,10343E-07	0,4372	145,65
0,3569	0,00547216	0,058	1,8284E-11	0,1607	233,71
0,01958	0,0167047	0,2033	4,31733E-07	0,1191	131,48
0,03244	0,00823623	0,1578	1,06507E-12	0,4649	261,65
0,148	0,00802224	0,0782	3,27047E-07	0,297	134,55
0,3137	0,00549191	0,0599	2,28278E-07	0,7652	138,34
0,01297	0,0147306	0,2499	1,74561E-07	0,2629	141,29
0,2607	0,00544061	0,0632	2,77262E-07	0,0420901	136,25
0,2555	0,00591995	0,064	3,95134E-07	0,7403	133,07
0,2244	0,00630708	0,0668	1,28103E-07	0,7833	143,67
0,3633	0,00530438	0,0577	4,73059E-11	0,845	223,84

0,2042	0,00629715	0,0695	3,32745E-08	0,686099	157,19
0,06692	0,00885138	0,1106	2,08938E-07	0,763901	139,08
0,1931	0,00650188	0,07	5,67258E-08	0,9207	150,87
0,184	0,00628848	0,0714	1,43603E-09	0,5081	188,88
0,307	0,00553787	0,0601	3,66622E-07	0,1161	133,58
0,5143	0,00523984	0,0557	2,07845E-07	0,0422202	139,29
0,3277	0,0067914	0,0199	1,10343E-07	0,9046	145,65
0,3569	0,00547216	0,0194	1,8284E-11	0,6863	233,71
0,01956	0,0167047	0,0674	4,31733E-07	0,4327	131,48
0,03257	0,00823623	0,0524	1,06507E-12	0,01071	261,65
0,148	0,00802224	0,0262	3,27047E-07	0,8085	134,55
0,3135	0,00549191	0,0201	2,28278E-07	0,1811	138,34
0,013	0,0147306	0,0825	1,74561E-07	0,6159	141,29
0,2607	0,00544061	0,0211	2,77262E-07	0,8416	136,25
0,2556	0,00591995	0,0214	3,95134E-07	0,2133	133,07
0,2245	0,00630708	0,0223	1,28103E-07	0,7154	143,67
0,3629	0,00530438	0,0193	4,73059E-11	0,006364	223,84
0,2044	0,00629715	0,0232	3,32745E-08	0,0568997	157,19
0,0669	0,00885138	0,037	2,08938E-07	0,5488	139,08
0,1928	0,00650188	0,0235	5,67258E-08	0,01685	150,87
0,1841	0,00628848	0,0239	1,43603E-09	0,9337	188,88
0,3072	0,00553787	0,0201	3,66622E-07	0,0464601	133,58
0,5142	0,00523984	0,0186	2,07845E-07	0,9815	139,29
0,3279	0,0067914	0,0204	1,10343E-07	0,716901	145,65
0,3569	0,00547216	0,0199	1,8284E-11	0,5427	233,71
0,01957	0,0167047	0,0693	4,31733E-07	0,608199	131,48
0,03256	0,00823623	0,0535	1,06507E-12	0,00310099	261,65
0,1482	0,00802224	0,0268	3,27047E-07	0,9946	134,55
0,3133	0,00549191	0,0205	2,28278E-07	0,1384	138,34
0,01288	0,0147306	0,0847	1,74561E-07	0,6511	141,29
0,2604	0,00544061	0,0217	2,77262E-07	0,7484	136,25
0,2558	0,00591995	0,0219	3,95134E-07	0,2206	133,07

0,2251	0,00630708	0,0228	1,28103E-07	0,8951	143,67
0,3625	0,00530438	0,0198	4,73059E-11	0,00408799	223,84
0,2046	0,00629715	0,0238	3,32745E-08	0,1055	157,19
0,06698	0,00885138	0,0379	2,08938E-07	0,4685	139,08
0,192	0,00650188	0,0241	5,67258E-08	0,0361102	150,87
0,1838	0,00628848	0,0245	1,43603E-09	0,5549	188,88
0,3079	0,00553787	0,0206	3,66622E-07	0,1748	133,58
0,5142	0,00523984	0,0191	2,07845E-07	0,5931	139,29
0,3278	0,0067914	0,0267	1,10343E-07	0,2835	145,65
0,3568	0,00547216	0,026	1,8284E-11	0,8582	233,71
0,0251	0,0454511	0,0792	4,69744E-06	0,2156	109,12
0,5058	0,0051599	0,0249	2,33637E-06	0,4316	114,78
0,01959	0,0167047	0,0906	4,31733E-07	0,745901	131,48
0,03248	0,00823623	0,0708	1,06507E-12	0,0760694	261,65
0,002037	0,0226212	0,2848	2,51151E-06	0,3226	114,46
0,3597	0,00551762	0,0261	4,81907E-06	0,5043	107,6
0,5104	0,00512668	0,0249	3,1567E-06	0,1064	111,9
0,1482	0,00802224	0,0351	3,27047E-07	0,665301	134,55
0,3221	0,00534652	0,0269	2,47648E-06	0,2661	114,74
0,1715	0,00745231	0,033	5,47314E-07	0,3616	128,65
0,09716	0,0082016	0,0422	4,10425E-06	0,921	108,94
0,4629	0,00523696	0,025	3,96588E-06	0,5241	109,98
0,3133	0,00549191	0,027	2,28278E-07	0,1073	138,34
0,2773	0,00860563	0,0278	7,19695E-07	0,4079	126,75
0,4531	0,00514647	0,0251	1,45525E-06	0,5011	119,8
0,2591	0,00588847	0,0285	2,40776E-06	0,8493	114,74
0,3746	0,00520724	0,0258	6,43688E-07	0,3307	127,78
0,4562	0,00519588	0,025	1,99226E-06	0,7212	117,14
0,4624	0,0051675	0,025	5,54023E-07	0,583001	129,63
0,103	0,0089572	0,0411	2,30041E-06	0,0483504	115,86
0,01289	0,0147306	0,1119	1,74561E-07	0,2123	141,29
0,1413	0,0118232	0,0359	2,13436E-06	0,2526	116,41

0,2605	0,00544061	0,0283	2,77262E-07	0,04583	136,25
0,2558	0,00591995	0,0287	3,95134E-07	0,0754102	133,07
0,2251	0,00630708	0,0298	1,28103E-07	0,7743	143,67
0,3626	0,00530438	0,0259	4,73059E-11	0,00758193	223,84
0,3177	0,0053782	0,0268	1,80548E-06	0,00885095	117,64
0,3071	0,00596416	0,0271	6,94393E-07	0,7012	127,29
0,2713	0,00720811	0,028	1,11551E-06	0,2754	122,29
0,1187	0,00759621	0,0388	2,26511E-06	0,904	115,05
0,4078	0,00515976	0,0253	2,65418E-06	0,3246	113,48
0,3039	0,0056781	0,027	4,56121E-06	0,2824	108,53
0,381	0,00528782	0,0256	8,23208E-07	0,2712	125,29
0,1361	0,00744654	0,0366	1,00922E-06	0,5924	123,35
0,3467	0,00539032	0,0262	2,5449E-06	0,7984	114,01
0,2045	0,00629715	0,0312	3,32745E-08	0,1623	157,19
0,4001	0,00543121	0,0255	9,48143E-07	0,7796	124,09
0,229	0,00674275	0,0296	4,73962E-06	0,8076	108,26
0,1432	0,00708759	0,0356	3,07171E-06	0,9885	112,79
0,06696	0,00885138	0,0496	2,08938E-07	0,2809	139,08
0,1922	0,00650188	0,0316	5,67258E-08	0,514	150,87
0,2956	0,0060781	0,0273	3,16088E-06	0,8241	112,13
0,3313	0,00541762	0,0266	4,75044E-06	0,1968	107,98
0,4475	0,00518301	0,025	4,12362E-06	0,6995	109,55
0,1837	0,00628848	0,032	1,43603E-09	0,8654	188,88
0,3085	0,00531236	0,027	4,87795E-07	0,3092	131,05
0,2502	0,00603038	0,0287	2,13305E-06	0,03093	115,75
0,2136	0,00672387	0,0303	3,86923E-06	0,9802	110,22
0,2981	0,00548864	0,0272	3,36101E-06	0,767499	111,32
0,126	0,0129749	0,0375	4,22478E-06	0,5707	109,13
0,3078	0,00553787	0,0269	3,66622E-07	0,5061	133,58
0,5143	0,00523984	0,025	2,07845E-07	0,6549	139,29
0,1561	0,00738691	0,0343	6,9643E-07	0,3778	127,04
0,328	0,0067914	0,029	1,10343E-07	0,1371	145,65

0,357	0,00547216	0,0282	1,8284E-11	0,2593	233,71
0,01958	0,0167047	0,0984	4,31733E-07	0,6296	131,48
0,03251	0,00823623	0,075	1,06507E-12	0,0467003	261,65
0,1481	0,00802224	0,0382	3,27047E-07	0,724499	134,55
0,3135	0,00549191	0,0291	2,28278E-07	0,4543	138,34
0,01286	0,0147306	0,1189	1,74561E-07	0,4492	141,29
0,2602	0,00544061	0,0308	2,77262E-07	0,0752905	136,25
0,2556	0,00591995	0,0311	3,95134E-07	0,6886	133,07
0,2251	0,00630708	0,0325	1,28103E-07	0,5464	143,67
0,3627	0,00530438	0,0281	4,73059E-11	0,1185	223,84
0,2045	0,00629715	0,0338	3,32745E-08	0,3442	157,19
0,06702	0,00885138	0,0538	2,08938E-07	0,8317	139,08
0,1921	0,00650188	0,0341	5,67258E-08	0,01034	150,87
0,1838	0,00628848	0,0348	1,43603E-09	0,4088	188,88
0,3079	0,00553787	0,0293	3,66622E-07	0,1031	133,58
0,5143	0,00523984	0,0271	2,07845E-07	0,8797	139,29
0,194699	0,0067914	0,008424	1,10343E-07	0,209506	145,65
0,334673	0,00547216	0,0069312	1,8284E-11	0,362865	233,71
0,0257773	0,0167047	0,0209182	4,31733E-07	0,600624	131,48
0,112898	0,00823623	0,0103593	1,06507E-12	0,0263572	261,65
0,27324	0,00579587	0,0073474	2,62457E-08	0,451561	160,31
0,117531	0,00802224	0,0101532	3,27047E-07	0,949087	134,55
0,344744	0,00549191	0,0069175	2,28278E-07	0,0483114	138,34
0,036838	0,0147306	0,017925	1,74561E-07	0,978223	141,29
0,336879	0,00544061	0,0069046	2,77262E-07	0,455867	136,25
0,274795	0,00591995	0,007417	3,95134E-07	0,518143	133,07
0,210525	0,00630708	0,0079965	1,28103E-07	0,00157474	143,67
0,382741	0,00530438	0,0067197	4,73059E-11	0,00055052	223,84
0,241366	0,00629715	0,0077842	3,32745E-08	0,510203	157,19
0,0949792	0,00885138	0,0111863	2,08938E-07	0,931836	139,08
0,190447	0,00650188	0,0083163	5,67258E-08	0,631985	150,87
0,216833	0,00628848	0,0079364	1,43603E-09	0,0211836	188,88

0,315665	0,00553787	0,0070337	3,66622E-07	0,00500714	133,58
0,427043	0,00523984	0,0066256	2,07845E-07	0,00060385	139,29
0,194712	0,0067914	0,0001494	1,10343E-07	0,31	145,65
0,334533	0,00547216	0,0001229	1,8284E-11	0,709999	233,71
0,025641	0,0167047	0,0003722	4,31733E-07	0,12	131,48
0,112301	0,00823623	0,0001844	1,06507E-12	0,69	261,65
0,274267	0,00579587	0,0001302	2,62457E-08	0,47	160,31
0,118142	0,00802224	0,0001798	3,27047E-07	0,44	134,55
0,344967	0,00549191	0,0001227	2,28278E-07	0,25	138,34
0,036755	0,0147306	0,0003182	1,74561E-07	0,780001	141,29
0,334775	0,00544061	0,0001226	2,77262E-07	0,56	136,25
0,275071	0,00591995	0,0001315	3,95134E-07	0,77	133,07
0,210178	0,00630708	0,0001421	1,28103E-07	0,0560003	143,67
0,381744	0,00530438	0,0001192	4,73059E-11	0,630001	223,84
0,240721	0,00629715	0,0001382	3,32745E-08	0,29	157,19
0,095115	0,00885138	0,0001982	2,08938E-07	0,37	139,08
0,191479	0,00650188	0,0001471	5,67258E-08	0,82	150,87
0,215426	0,00628848	0,000141	1,43603E-09	0,68	188,88
0,316608	0,00553787	0,0001246	3,66622E-07	0,5	133,58
0,426593	0,00523984	0,0001175	2,07845E-07	0,93	139,29
0,011195	0,0454511	0,099125	4,69744E-06	0,234179	109,12
0,0252933	0,0167047	0,057094	4,31733E-07	0,402264	131,48
0,101402	0,00823623	0,034021	1,06507E-12	0,0638675	261,65
0,132172	0,00802224	0,026458	3,27047E-07	0,302526	134,55
0,407645	0,00534652	0,019444	2,47648E-06	0,673572	114,74
0,142032	0,00745231	0,025067	5,47314E-07	0,774118	128,65
0,105556	0,0082016	0,033885	4,10425E-06	0,639424	108,94
0,466239	0,00523696	0,020096	3,96588E-06	0,424656	109,98
0,328597	0,00549191	0,018783	2,28278E-07	0,44301	138,34
0,113306	0,00860563	0,028054	7,19695E-07	0,827985	126,75
0,475048	0,00514647	0,01815	1,45525E-06	0,460208	119,8
0,262756	0,00588847	0,022535	2,40776E-06	0,791797	114,74

0,439283	0,00519588	0,017915	1,99226E-06	0,989636	117,14
0,110456	0,0089572	0,027148	2,30041E-06	0,239368	115,86
0,062586	0,0118232	0,036009	2,13436E-06	0,462619	116,41
0,310868	0,00544061	0,019125	2,77262E-07	0,589454	136,25
0,273327	0,00591995	0,020688	3,95134E-07	0,794842	133,07
0,205024	0,00630708	0,022462	1,28103E-07	0,203133	143,67
0,371096	0,0053782	0,018417	1,80548E-06	0,932397	117,64
0,264945	0,00596416	0,020517	6,94393E-07	0,589992	127,29
0,151405	0,00720811	0,024628	1,11551E-06	0,983039	122,29
0,480726	0,00515976	0,019921	2,65418E-06	0,770031	113,48
0,289544	0,0056781	0,019706	4,56121E-06	0,92809	108,53
0,383622	0,00528782	0,018456	8,23208E-07	0,945181	125,29
0,165006	0,00744654	0,024649	1,00922E-06	0,505075	123,35
0,370758	0,00539032	0,018295	2,5449E-06	0,254225	114,01
0,230419	0,00629715	0,024233	3,32745E-08	0,72097	157,19
0,186575	0,00650188	0,022098	5,67258E-08	0,231947	150,87
0,237455	0,0060781	0,020726	3,16088E-06	0,489726	112,13
0,380153	0,00541762	0,018793	4,75044E-06	0,949239	107,98
0,472839	0,00518301	0,01804	4,12362E-06	0,680373	109,55
0,212448	0,00628848	0,021593	1,43603E-09	0,667327	188,88
0,183913	0,00672387	0,023277	3,86923E-06	0,262472	110,22
0,0515458	0,0129749	0,041199	4,22478E-06	0,443598	109,13
0,343199	0,00553787	0,018954	3,66622E-07	0,780497	133,58
0,426366	0,00523984	0,018793	2,07845E-07	0,822265	139,29
0,127789	0,00738691	0,026347	6,9643E-07	0,796199	127,04
0,210577	0,0067914	0,03769	1,10343E-07	0,611001	145,65
0,330063	0,00547216	0,030829	1,8284E-11	0,439821	233,71
0,520333	0,0051599	0,028994	2,33637E-06	0,46762	114,78
0,020777	0,0167047	0,107223	4,31733E-07	0,380548	131,48
0,288345	0,00579587	0,032774	2,62457E-08	0,254385	160,31
0,404839	0,00534652	0,030102	2,47648E-06	0,946625	114,74
0,13897	0,00745231	0,041726	5,47314E-07	0,799935	128,65

0,455472	0,00523696	0,031516	3,96588E-06	0,69501	109,98
0,328105	0,00549191	0,031419	2,28278E-07	0,46097	138,34
0,264341	0,00588847	0,035287	2,40776E-06	0,95264	114,74
0,039156	0,0147306	0,084818	1,74561E-07	0,317443	141,29
0,06368	0,0118232	0,059623	2,13436E-06	0,547089	116,41
0,311702	0,00544061	0,031747	2,77262E-07	0,501534	136,25
0,204321	0,00630708	0,035649	1,28103E-07	0,27504	143,67
0,364439	0,00530438	0,030195	4,73059E-11	0,747632	223,84
0,373765	0,0053782	0,029991	1,80548E-06	0,495898	117,64
0,362648	0,0054735	0,031023	2,71919E-06	0,509354	113,69
0,144741	0,00720811	0,040494	1,11551E-06	0,597324	122,29
0,116499	0,00759621	0,045183	2,26511E-06	0,911439	115,05
0,480443	0,00515976	0,030639	2,65418E-06	0,767125	113,48
0,285731	0,0056781	0,031987	4,56121E-06	0,976711	108,53
0,37954	0,00528782	0,030109	8,23208E-07	0,860421	125,29
0,160864	0,00744654	0,040222	1,00922E-06	0,496581	123,35
0,229042	0,00629715	0,038635	3,32745E-08	0,615072	157,19
0,355488	0,00543121	0,030335	9,48143E-07	0,450894	124,09
0,202007	0,00674275	0,036638	4,73962E-06	0,987295	108,26
0,175047	0,00708759	0,038484	3,07171E-06	0,55341	112,79
0,186442	0,00650188	0,037168	5,67258E-08	0,846225	150,87
0,381659	0,00541762	0,030366	4,75044E-06	0,931152	107,98
0,214199	0,00628848	0,035835	1,43603E-09	0,748264	188,88
0,224295	0,00603038	0,034562	2,13305E-06	0,803486	115,75
0,186018	0,00672387	0,037325	3,86923E-06	0,891952	110,22
0,323625	0,00548864	0,03112	3,36101E-06	0,905735	111,32
0,051681	0,0129749	0,06411	4,22478E-06	0,863646	109,13
0,424831	0,00523984	0,03028	2,07845E-07	0,925198	139,29
0,125997	0,00738691	0,043844	6,9643E-07	0,740183	127,04
0,195643	0,0067914	0,024124	1,10343E-07	0,366087	145,65
0,00809	0,0454511	0,097143	4,69744E-06	0,904877	109,12
0,520031	0,0051599	0,018551	2,33637E-06	0,435611	114,78

0,024675	0,0167047	0,066651	4,31733E-07	0,661206	131,48
0,279549	0,00579587	0,02075	2,62457E-08	0,698163	160,31
0,134273	0,00802224	0,028015	3,27047E-07	0,30509	134,55
0,415277	0,00534652	0,019431	2,47648E-06	0,389339	114,74
0,14256	0,00745231	0,026817	5,47314E-07	0,801683	128,65
0,097634	0,0082016	0,033568	4,10425E-06	0,97126	108,94
0,442877	0,00523696	0,020159	3,96588E-06	0,794503	109,98
0,33351	0,00549191	0,020013	2,28278E-07	0,724461	138,34
0,468515	0,00514647	0,018688	1,45525E-06	0,183332	119,8
0,258606	0,00588847	0,022777	2,40776E-06	0,986401	114,74
0,442409	0,00519588	0,018715	1,99226E-06	0,331374	117,14
0,467321	0,0051675	0,018806	5,54023E-07	0,0928603	129,63
0,103835	0,0089572	0,031517	2,30041E-06	0,466475	115,86
0,032817	0,0147306	0,055904	1,74561E-07	0,80045	141,29
0,064621	0,0118232	0,038592	2,13436E-06	0,232326	116,41
0,318695	0,00544061	0,020218	2,77262E-07	0,29444	136,25
0,223458	0,00630708	0,022859	1,28103E-07	0,193311	143,67
0,371332	0,00530438	0,019368	4,73059E-11	0,0228544	223,84
0,36894	0,0053782	0,019162	1,80548E-06	0,997312	117,64
0,387353	0,0054735	0,019781	2,71919E-06	0,49954	113,69
0,166282	0,00720811	0,025856	1,11551E-06	0,851934	122,29
0,485329	0,00515976	0,019599	2,65418E-06	0,364369	113,48
0,271097	0,0056781	0,020576	4,56121E-06	0,533909	108,53
0,399655	0,00528782	0,019178	8,23208E-07	0,134126	125,29
0,14596	0,00744654	0,025919	1,00922E-06	0,856084	123,35
0,364959	0,00539032	0,019409	2,5449E-06	0,241167	114,01
0,230436	0,00629715	0,024801	3,32745E-08	0,564481	157,19
0,334399	0,00543121	0,019446	9,48143E-07	0,797566	124,09
0,157812	0,00708759	0,024962	3,07171E-06	0,206739	112,79
0,090808	0,00885138	0,033215	2,08938E-07	0,579486	139,08
0,191022	0,00650188	0,023664	5,67258E-08	0,429073	150,87
0,236383	0,0060781	0,021784	3,16088E-06	0,699977	112,13

0,356783	0,00541762	0,019407	4,75044E-06	0,417897	107,98
0,469322	0,00518301	0,018724	4,12362E-06	0,581735	109,55
0,201146	0,00628848	0,022884	1,43603E-09	0,376588	188,88
0,23575	0,00603038	0,022058	2,13305E-06	0,862701	115,75
0,201838	0,00672387	0,023811	3,86923E-06	0,517315	110,22
0,057118	0,0129749	0,042091	4,22478E-06	0,45559	109,13
0,396325	0,00523984	0,019329	2,07845E-07	0,901974	139,29
0,120505	0,00738691	0,027708	6,9643E-07	0,364091	127,04

©2024 Wang X.J. et al.
